# Undiagnosed Sacrococcygeal Fistulas: A Case Report

**DOI:** 10.7759/cureus.100561

**Published:** 2026-01-01

**Authors:** Marta De Figueiredo Martins, Pedro Valério, Ana Paula Pinheiro

**Affiliations:** 1 Family Medicine, Unidade de Saúde Familiar Viseu-Cidade, Unidade Local de Saúde Viseu Dão-Lafões, Viseu, PRT

**Keywords:** disease progression, fistula, health behavior, personal autonomy, shame

## Abstract

The clinical presentation of fistulas can include persistent drainage, pain or swelling. In the perianal, gluteal and sacrococcygeal areas, differential diagnosis may include perianal fistula, malignancy, inflammatory bowel disease or pilonidal disease. Lack of treatment can lead to disease progression. Patient appraisal of symptoms plays an important role in clinical outcomes, a role possibly shared with the shame of exposing symptoms. A behavior of avoidance and withholding of information can directly impact clinical outcomes by not seeking medical help and delays in treatment. This case describes a 74-year-old man who, during a routine visit, reports complaints of purulent drainage in the sacrococcygeal region. This complaint was more than fifty years old and had been undervalued until now, according to the patient, because it lacked significant symptoms and the embarrassment of exposing the condition. The symptoms worsened in the previous two weeks, leading him to report the complaints. On physical examination, extensive fistulous disease in the sacrococcygeal region with purulent discharge was observed. Antibiotic therapy was started (amoxicillin and clavulanic acid - 875 mg plus 125 mg every 12 hours), given the worsening of symptoms described. The patient was referred to a surgical consultation where antibiotic therapy with metronidazole (500 mg every 12 hours) was added, and a targeted workup was performed but proved non-definitive. After considering the risks and benefits of an intervention, the patient opted for a non-invasive expectant attitude, preserving his autonomy in medical decisions.

## Introduction

Fistulas can be defined as an epithelial-lined communication between two structures [1-4]. The most common fistulas are of cryptoglandular origin, arising from infection of anal glands, but they can also arise in a disease-associated setting [2,3]. The clinical presentation consists of persistent drainage, pain, swelling with or without systemic symptoms in more complicated forms (abscess or sepsis) [1,2,4,5]. Targeted investigation of a fistula begins with patient history and physical examination, which can be complemented by imaging to determine the anatomy of the tract and type of fistula [1,3,5-7]. Management may include surgical approaches and medical therapies, according to the underlying etiology [2-4,7]. In the perianal, gluteal and sacrococcygeal areas, differential diagnosis may include perianal fistula, malignancy, inflammatory bowel disease or pilonidal disease [1,4,6,8].

Lack of treatment can lead to disease progression, reinforcing the need for timely management [1]. The patient may be reluctant to search for treatment for various reasons, including not knowing where to go or feelings of embarrassment [9].

Fistulizing disease, in general, is known for its psychological impact, which has been studied as a factor in its outcome [1,10]. Shame, on the other hand, is another powerful factor in patient care and, as Frank Davidoff highlights in his article [11], patients may often view their illnesses as defects or inadequacies and believe that contact with health services can lead to humiliating exposure. The result can be a behavior of avoidance and withholding of information, which has a direct impact on clinical outcomes by not seeking medical help and delays in treatment [11,12].

In parallel, patient appraisal of symptoms also plays an important role in clinical outcomes by interfering with prediagnosis intervals and longer intervals until referrals have been linked to more advanced findings [13,14]. This case report proves to be an example of this dynamic.

## Case presentation

**Figure 1:**
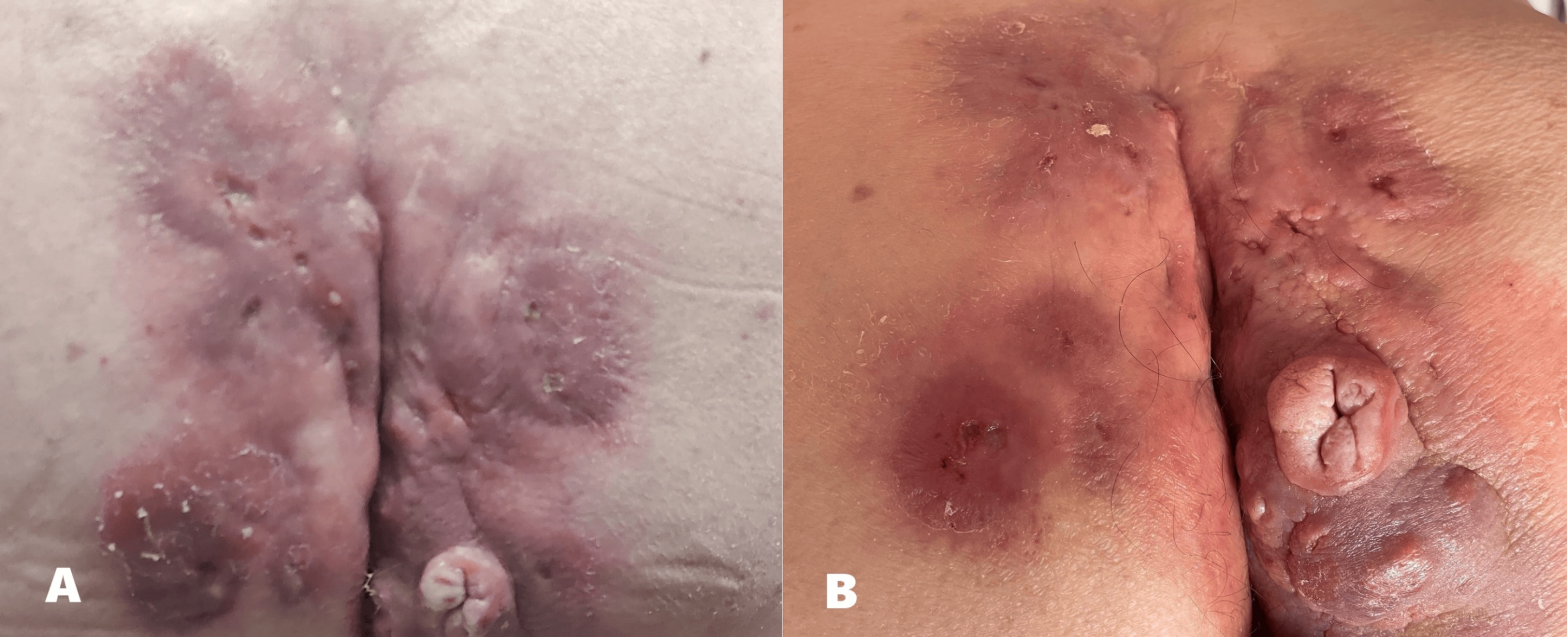
Findings in the first visit (A), and after 6 months (B).

A 74-year-old man, during a routine hypertension monitoring visit, reports complaints of purulent drainage in the sacrococcygeal region, seeking help in managing this problem. When delving into the clinical history of this complaint, we discovered that it was more than 50 years long. The patient admitted having been evaluated in the very beginning of the clinical course, and that surgery was suggested, but not performed. He could not give further details about the reasoning behind this and stated that he had been undervaluing this condition until now, because it lacked significant symptoms, and he was embarrassed of exposing the condition. After the initial and momentary evaluation described in the early days of the condition, the patient simply managed these symptoms at home for 50 years. With the help of his wife, the management was conducted by applying clean cloths that were washed and changed daily and regional hygiene. He further explained that the symptoms had worsened in the previous two weeks, becoming bothersome, which led him to report the complaints during the visit. He denied any concomitant new systemic signs or symptoms. On physical examination, we found extensive fistulizing disease in the sacrococcygeal and gluteal region with purulent discharge. The skin appeared macerated, and we could observe both active fistulas and scarring from previous ones (Figure 1A).

**Figure 2:**
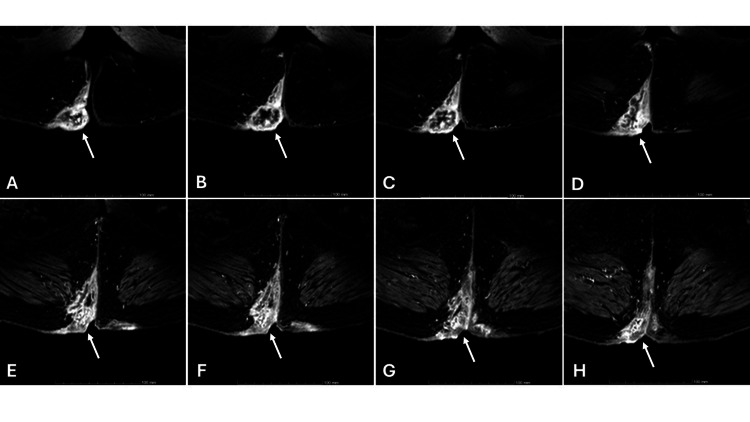
Pelvic magnetic resonance imaging of the patient described in the clinical case is shown in images A through H.

Antibiotic therapy was started with amoxicillin and clavulanic acid (875 mg plus 125 mg every 12 hours), given the worsening of symptoms described. The patient was referred to a surgical consultation, where a directed workup was performed and antibiotic therapy with metronidazole (500 mg every 12 hours) was added. The patient was submitted to a colonoscopy, magnetic resonance imaging (MRI) and tumour markers. This workup proved, however, non-definitive: colonoscopy found no aspects of inflammatory bowel disease, cancer markers were negative, and, in the end, the MRI described a lesion with a non-contrast-enhancing central area (suggesting a necrotic or abscessed nature), however, an underlying tumoral etiology was only possible to exclude with biopsy. Figure 2 shows the MRI findings.

**Figure 3:**
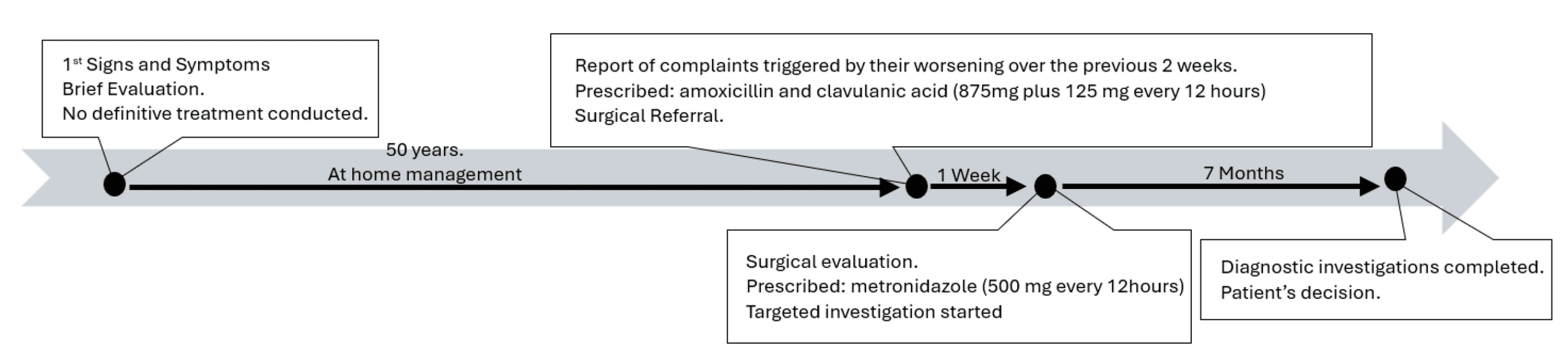
Brief chronology of the events and their management.

After completing the diagnostic workup and reviewing the detailed findings with his family, the patient decided not to pursue an invasive approach and opted for a non-invasive expectant attitude and surveillance. Figure 3 illustrates the chronology of the events and their management.

## Discussion

This case demonstrates several important points. Firstly, we cannot ignore how patients’ appraisal of symptoms impacted disease evolution through time. The patient confessed that he considered the condition lacked significant symptoms. This fact may be difficult for us to relate to, given the physical findings, but we can understand that an indolent evolution of persistent physical symptoms may contribute to the integration of these symptoms as part of a normalized scenario [13,15].

We did not observe how the symptoms first presented, given their distant relation to the current time, so we rely only on the description given by the patient, but, even so, we are certain that the exuberant findings are a far cry from 50 years ago. Even though an investigation was performed to unveil the origin of this exuberant finding, the result was not conclusive. We could consider perianal fistula, a pilonidal disease, an underlying inflammatory bowel disease or malignancy, or perhaps a combination of more than one etiology or another hypothesis altogether [1,4,6,8].

A perianal fistula connects perianal skin with the anal canal and is commonly associated with anorectal abscess, but it can also occur in the setting of inflammatory bowel disease or malignancy [4,5]. In this case, fistulous tracts were in the sacrococcygeal area, somewhat distant from the anal canal. Pilonidal disease is an acquired disease and manifests as midline pits in the gluteal cleft or sacrococcygeal area, with persistent drainage and subcutaneous tracts in its chronic form. Most courses cephalad, but caudal direction is also possible and may be confused with anorectal fistulas, making evaluation for concomitant fistulous disease important [6,8,16,17]. Inflammatory bowel disease usually includes specific features and symptoms that were not present in the patient [3,18].

We can theorize that, given the evolution, a colonoscopy with no signs of inflammatory bowel disease, negative tumour markers, and considering that the fistulous tracts were in the sacrococcygeal area, somewhat distant localization from the anal margin, a long-standing, not treated pilonidal disease may be a strong contender [1,4-6,8,16,18]. We, however, cannot ignore the MRI report not excluding underlying tumoral etiology.

Risk factors for pilonidal disease include a sedentary lifestyle, obesity, repetitive trauma and hirsutism [6,17]. Disease presentation can vary from asymptomatic pits to chronic sinuses, and one of the factors related to this is its duration, in this case, a particularly prolonged one. Chronic form is related to significant morbidity [17].

Secondly, this case reminds us of facts beyond the clinical ones. Shame may cause patients to withhold from presenting their complaint, which was confessed by the patient and recognized as an important factor in the evolution of symptoms [11,12]. As described in the introduction, feelings of embarrassment are one of the reasons patients may be reluctant to seek treatment [9]. On the other hand, patient autonomy in medical decisions proved to be an important point in deciding how far to go in the diagnostic evaluation, since the patient opted for a non-invasive expectant attitude and surveillance - a decision based on the existing data and outcome of an intervention relayed [19]. To be noted, this decision was reached in a combined reflection with his family. We should remember that it was his wife who helped him with wound care during all these years, and even though in the end we discovered that, for years, she had been insisting with her husband for an observation, she respected his timing and never confessed his hidden symptoms.

## Conclusions

Fistulas can have a heterogeneous origin and their symptoms can include: persistent drainage, pain, swelling with or without systemic symptoms. Investigation should begin with patient history and physical examination and be complemented with imaging. This case report describes a male with a particularly prolonged clinical scenario of fistulizing disease of the sacrococcygeal area. It shows us how patients’ appraisal of symptoms can impact their evolution through time, leading to exuberant and rare presenting clinical cases. In this case, the outcome might be conditioned by the extensive presentation of the fistulizing disease after a 50-year long evolution, since lack of treatment can lead to disease progression, reinforcing the relevance of timely management. However, in this case, we must consider facts beyond the clinical ones. On one hand, this case illustrates how shame may cause patients to withhold from presenting their complaint, reinforcing how feelings of embarrassment have been associated with reluctance to seek treatment. On the other hand, how patient autonomy in medical decisions must not be forgotten when achieving a patient-centred approach. In this context, it was the patient’s autonomy in medical decision-making that dictated how far we could go in medical interventions.
